# Matrifibrocytes Redefine Cardiac Fibrosis: From Terminal Differentiation to Translational Modulation

**DOI:** 10.1111/jcmm.71174

**Published:** 2026-05-13

**Authors:** Zhentao Zhang, Hua Zhu

**Affiliations:** ^1^ Department of Surgery, Davis Heart and Lung Research Institute The Ohio State University Wexner Medical Center Columbus Ohio USA

**Keywords:** cardiac fibroblast, fibrosis, matrifibrocyte, myofibroblast, scar maturation

## Abstract

Cardiac fibrosis is increasingly recognized as a dynamic program that resolves into a terminal fibroblast fate, the matrifibrocyte, rather than a persistent myofibroblast state. Lineage‐tracing and single‐cell studies reveal that matrifibrocytes arise from activated myofibroblasts during late scar maturation, lose *α*‐SMA and proliferative capacity, and adopt a cartilage‐like ECM program (e.g., *Comp*, *Chad*, *Thbs4*, *Sfrp2*). Functionally, they stabilize collagen architecture, sustain tensile strength and shape an immune‐quiescent, angiogenesis‐restrained microenvironment. We synthesize evidence suggesting that TGF‐*β* restraint (e.g., *Smad7*), matrix mechanics (YAP/TAZ), Wnt attenuation (*Sfrp2*), *Comp–Notch3* feedback, and *Thbs4–Atf6α* ER‐stress adaptation converge to establish or maintain this terminal state. We further summarize disease contexts beyond infarction, including pressure overload and valvular disease, where matrifibrocyte‐like programs emerge and may contribute to chronic stiffening. Finally, we outline translational strategies that either accelerate matrifibrocyte maturation to secure scars after acute injury or reset terminal states via pharmacology or direct reprogramming to regress fibrosis. Framing matrifibrocytes as an adjustable endpoint reconciles structural integrity with functional recovery and highlights actionable checkpoints for precision anti‐fibrotic therapy.

## 1 Introduction

Cardiac fibrosis is a hallmark of diverse heart diseases and a central driver of heart failure, arrhythmogenesis and adverse remodelling [1]. It reflects excessive extracellular matrix (ECM) deposition and remodelling triggered by myocardial infarction (MI), chronic pressure overload, metabolic stress or aging. Resident cardiac fibroblasts (CFs) are the principal effectors: upon injury they activate into myofibroblasts (MFs), highly contractile, ECM‐secreting cells essential for early tissue integrity and rupture prevention [2]. This initially adaptive response becomes maladaptive when persistent, producing stiffening and diastolic dysfunction, defining features of heart failure [3, 4, 5, 6]. While mechanisms of CF activation and MF differentiation are increasingly understood [7, 8, 9, 10, 11, 12, 13], the resolution phase and the basis of scar homeostasis remained unclear. The prevailing view, MF apoptosis or reversion to quiescence, has been challenged by lineage‐tracing evidence showing a substantial fibroblast‐derived population that persists in mature scars with a distinct, matrix‐preserving phenotype.

In 2018, Fu et al. addressed this gap using fibroblast‐specific lineage tracing and transcriptomics, revealing that late‐phase post‐MI MF transition into a terminally differentiated state [14]. These cells, termed matrifibrocytes (MFCs), are non‐contractile, low‐proliferative, and defined by a cartilage‐like ECM gene signature, for example *Comp*, *Chad*, and *Cilp*. Identifying MFCs established an unrecognized branch of the fibroblast lineage that emerges during scar maturation and reframed how resolution and maintenance of cardiac fibrosis are conceptualized [14].

This review integrates cellular, molecular and systems‐level advances. We outline the fibroblast lifecycle, from quiescent CFs to activated MFs to MFCs, emphasizing their temporal and functional hierarchy in repair. We then define MFC roles in scar stabilization and myocardial homeostasis, dissect the signalling and regulatory mechanisms that consolidate this fate and consider context‐specific influences. Finally, we synthesize a translational perspective: guiding this terminal state to control fibrosis, resolving pathological ECM accumulation without compromising structural integrity. Collectively, emerging concepts position MFC biology as a central paradigm linking tissue repair, fibrosis resolution and long‐term myocardial stability.

## 2 The Fibroblast Lifecycle: From Quiescence to Matrifibrocyte

The identification of MFCs refines the traditional activation paradigm. CFs follow a coordinated trajectory that culminates in this terminal state. Understanding MFCs therefore requires positioning them within the full lifecycle from quiescence, through transient MFs, to terminal differentiation.

### 2.1 Quiescent Fibroblasts: Sentinels of Myocardial Structure

**FIGURE 1:**
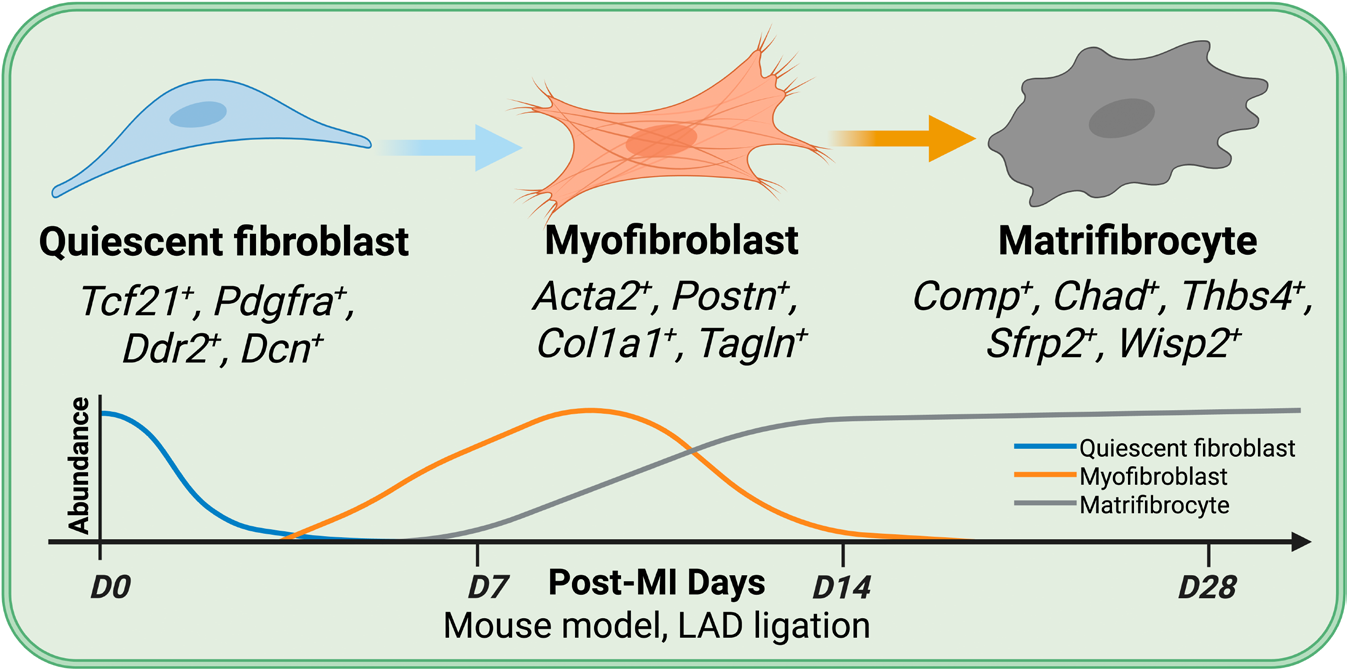
Temporal dynamics of fibroblast states after myocardial infarction. After myocardial injury, quiescent CFs (*Tcf21* ^+^, *Pdgfra* ^+^, *Ddr2* ^+^, and *Dcn*+) rapidly activate into *α*‐smooth muscle actin–positive (*Acta2* ^+^) MFs that produce ECM to stabilize the injured myocardium. MFs dominate during the early reparative phase (day 3–7 post‐MI) but progressively decline by day 14 as the wound matures. A subset of fibroblast‐lineage cells subsequently transitions into a terminal, non‐contractile state known as the MFC, characterized by the loss of stress fibres and upregulation of cartilage‐like ECM genes (*Comp*, *Chad*, *Thbs4,* and *Sfrp2*). The lower panel illustrates the relative temporal abundance of each cell population. Quiescent CFs rapidly decrease after injury (blue), MFs peak during early scar formation and resolve by day 14 (orange), while MFCs emerge around day 10 and persist as the dominant fibroblast population in the mature scar (day 14–28) (grey).

In the healthy adult heart, quiescent CFs typically comprise under 20% of non‐myocytes [15] and act as primary custodians of ECM homeostasis. They maintain myocardial structure through balanced collagen synthesis, regulated ECM turnover, and secretion of matricellular proteins [5, 16], thereby supporting tissue compliance, cardiomyocyte electromechanical coupling, and the coronary microvasculature [15]. Phenotypically, resting CFs express lineage and structural markers including *Tcf21*, *Pdgfra*, *Ddr2* and *Dcn*, but lack the contractile apparatus that characterises activated cells [17, 18] (Figure 1). Poised to respond to mechanical strain, neurohormonal cues or damage‐associated signals, they rapidly initiate reparative transcriptional programmes, marking the first step toward fibrogenesis.

### 2.2 Myofibroblasts: The Contractile Effectors of Scar Formation

Activation of quiescent CFs yields MFs, the principal effectors of early scar formation [2]. This conversion is driven by transforming growth factor‐*β* (TGF‐*β*), tension within provisional ECM, and neurohumoral cues such as angiotensin II [19, 20, 21]. MFs are defined by assembly of *α*‐smooth muscle actin (*α*‐SMA, encoded by *Acta2*) into stress fibres and by a profibrotic secretory program rich in type I and type III collagens and fibronectin [22, 23, 24, 25]. They upregulate *Postn*, *Col1a1*, *Tagln* and related matrix genes, generating contractile forces that promote wound contraction and stabilize the nascent scar, thereby providing tensile strength to the injured ventricle [26]. During activation, metabolism shifts toward glycolysis with increased oxidative stress [27, 28], supporting high contractile output. Key transcriptional drivers include SRF [29], MRTF‐A [30, 31, 32], *Smad3* and YAP/TAZ (herein TAZ refers to the transcriptional coactivator with PDZ‐binding motif encoded by WWTR1 per HGNC (HUGO Gene Nomenclature Committee) nomenclature, distinct from Tafazzin which shares the TAZ abbreviation) [33, 34]. After MI, MF abundance rises and typically peaks around day 7 to day 10 with concomitant induction of *Acta2*, *Postn*, *Col1a1* and *Tagln* [14, 26]. This phase is essential yet metabolically demanding and should be time limited; persistence drives pathological stiffening and diastolic dysfunction. Resolution of the MF state sets the stage for terminal remodelling, during which a substantial subset of MF‐derived cells transitions to matrifibrocytes that sustain matrix architecture (Figure 1).

### 2.3 Matrifibrocytes: A Terminal Fate in Fibroblast Evolution

Lineage‐tracing studies show that MFs need not universally undergo apoptosis; instead, many transition into a distinct, long‐lived fate, the MFC [14]. As scars mature, MFCs accumulate and predominate by approximately day 28, while the MF program tapers. This is not reversion to quiescence, but terminal differentiation characterised by loss of contractility (e.g., downregulation of *Acta2* and *Tagln*) and acquisition of a cartilage‐like ECM program including *Comp*, *Chad*, *Thbs4*, *Sfrp2* and *Wisp2*. In this state, cells exhibit low proliferative capacity and a non‐contractile, matrix‐preserving phenotype that stabilises collagen architecture and supports long‐term scar integrity (Figure 1).

Together, this lifecycle, from quiescent CFs to transient MFs to terminal MFCs, provides the context for the mechanisms and checkpoints dissected in the following sections.

## 3 Origin and Phenotypic Characteristics of the Matrifibrocyte

The discovery of the MFC has redefined the terminal fate of CFs. Its precise cellular origin, dynamic emergence, and distinct molecular phenotype collectively form the foundation for identifying and understanding this unique fibroblast state.

### 3.1 Defined Cellular Origin: Derived From Myofibroblasts

Lineage tracing offers the most direct evidence. Using *Tcf21* ^*MCM/*+^; *R26* ^*EGFP*^, *Postn* ^*MCM/*+^; *R26* ^*EGFP*^ *and Acta2* ^*CreERT2*^; *R26* ^*EGFP*^, Fu et al. showed that long‐lived fibroblast‐lineage cells persisting in mature infarct scars arise from activated fibroblasts or MFs [14]. These fate‐mapped cells initially express *α*‐SMA and Postn, then progressively lose contractile features while acquiring a transcriptional program enriched for cartilage‐associated ECM genes such as *Comp* and *Chad* [14]. Thus, MFCs represent a differentiation endpoint of activated fibroblasts rather than a separate lineage or a reversion to quiescence, challenging the view that MFs uniformly undergo apoptosis after repair.

### 3.2 Temporal Dynamics of Differentiation

MFC formation follows the phases of post‐infarction healing. In mouse MI, MFs dominate days 3 to 7, coincident with peak ECM deposition and wound contraction [14]. Beginning around day 10, a subset transitions: *α*‐SMA declines, stress fibres disassemble, and *Comp* and *Chad* increase. By the late reparative phase, these cells persist as a stable population embedded in the mature scar with a transcriptome distinct from quiescent and activated CFs [14, 35]. This temporally restricted conversion delineates MFC formation as an active, programmed differentiation event rather than a passive consequence of declining fibrotic activity. It marks the physiological shift of the scar from a dynamic, matrix‐producing environment toward a structurally consolidated and homeostatic state.

### 3.3 Distinct Phenotypic and Molecular Features

MFCs are defined by a constellation of molecular and cellular traits that sharply distinguish them from their MFs precursors. The most conspicuous transition is the downregulation or complete loss of *α*‐SMA and the dissolution of stress fibres, reflecting the withdrawal from contractile activity and commitment to matrix stabilization [14]. Transcriptomic analyses indicate markedly reduced expression of cell‐cycle regulators and proliferation markers, identifying MFCs as post‐mitotic, terminally differentiated cells with long‐term persistence within the scar [13, 14, 26]. MFCs express a specialized gene program reminiscent of dense connective tissues such as cartilage and tendon. Core markers include *Comp* (Cartilage Oligomeric Matrix Protein) [14], *Chad* (Chondroadherin) [14], *Wisp2* (WNT1‐inducible signalling pathway protein 2) [36], *Thbs4* (Thrombospondin‐4) [35, 37], *Sfrp2* (Secreted Frizzled‐Related Protein 2) [36, 38] and *Clip2* (CAP‐Gly domain‐containing linker protein 2) [13, 14]. The coordinated up‐regulation of these genes establishes a molecular identity distinct from all other cardiac cell types, including quiescent CFs, MFs, endothelial cells and immune populations, and underscores their specialized role in maintaining the structural integrity of the mature scar. As *α*‐SMA and Tagln decline, cells adopt rounded or stellate morphologies with low mechanical tension.

Collectively, these findings define the MFC as a terminal, post‐mitotic fibroblast state that arises in a temporally regulated manner from activated MFs and exhibits a unique molecular identity tailored for long‐term matrix maintenance. Having established its cellular origin, differentiation timeline and defining phenotypic features, the next step is to understand how these cells contribute functionally to scar stability and myocardial homeostasis.

## 4 Functional Roles of Matrifibrocytes

The emergence of MFCs represents the terminal phase of the cardiac wound‐healing program, in which the heart transitions from an active reparative environment to a mechanically stable scar. Far from being inert remnants of fibrosis, MFCs execute specialized structural and regulatory functions essential for long‐term tissue integrity.

### 4.1 Maintenance of Scar Integrity

Functional interrogation of fibroblast‐lineage cells during the late healing phase has demonstrated that MFCs are indispensable for the preservation of scar architecture. In the seminal Fu et al. study, selective ablation of lineage‐traced fibroblasts within the mature infarct scar using localized cryoinjury resulted in scar thinning, ventricular wall rupture and acute mortality, providing direct evidence that these MFCs are essential for structural preservation of the infarcted myocardium [14]. By persisting within the scar, these long‐lived fibroblast‐lineage matrifibrocytes maintain mechanical cohesion of the infarcted wall after myofibroblasts resolve, as ablation during late healing precipitates loss of scar integrity and wall failure.

### 4.2 Structural Remodelling and ECM Cross‐Linking

Histological analyses from 3 to 28 days after myocardial infarction revealed a progressive accumulation, condensation, and alignment of collagen fibres within the infarcted area, indicating continuous matrix maturation as the scar evolved from a loose fibrillar network to a densely organized structure [14]. This process temporally coincided with the transition of *α‐SMA*–positive MFs into *α‐SMA*–negative MFCs, suggesting that MFC differentiation is mechanistically linked to ECM stabilization. Transcriptomic profiling of lineage‐traced fibroblasts further revealed sustained enrichment of genes involved in collagen fibrillogenesis, cross‐linking, and ECM organization during this late phase [14, 39]. The upregulation of *Comp*, *Chad*, *Thbs4* and *Sfrp2* reflects a specialized biosynthetic program reminiscent of cartilage and tendon, promoting collagen fibre alignment and intermolecular bridging [14]. In functional terms, *Comp* promotes collagen fibre alignment and intermolecular bridging, *Chad* supports fibril anchoring and organization, and *Dpt* augments fibril spacing and order [14]. Matrix cross‐linking by the lysyl oxidase (*Lox*) family consolidates tensile strength; perturbations, such as *postn* deficiency or *Lox* inhibition delay loss of *α*‐SMA and prolong the myofibroblast state, underscoring the requirement for cross‐linking in scar consolidation [26]. Collectively, these findings position MFCs as the key executors of ECM remodelling that transforms a provisional wound matrix into a stable, load‐bearing scar.

### 4.3 Regulation of the Post‐Injury Microenvironment

MFCs upregulate *Thbs4*, which engages *Atf6α* and enhances endoplasmic reticulum proteostasis, thereby supporting a sustained secretory program in the mature scar. Through this program, MFC‐derived matricellular mediators help establish a low‐inflammation, anti‐angiogenic niche. Transcriptomic and secretome data show high *Thbs4*, *Dpt* and other mediators that modulate macrophage polarization and angiogenic signalling [14, 36]. *Thbs4* dampens inflammatory cytokines and restrains neovascularization, whereas *Dpt* improves fibril organization and favours a quiescent macrophage phenotype [36]. In angiotensin II–induced hypertrophy, *Cilp* ^+^ */Thbs4* ^+^ fibroblasts emerge as dominant non‐contractile ECM remodelers with downregulation of inflammatory and angiogenic ligands, mirroring MFC‐like programs [35]. Converging pathways consolidate this terminal state, including restraint of TGF‐*β* signalling with induction of *Smad7*, attenuation of Wnt signalling through *Sfrp2*, and *Comp–Notch3* coupling that reinforces matrix‐preserving programs; *Wisp2* is also enriched in late fibroblasts in several datasets and associates with resolution‐phase transcriptional states. Relative to activated myofibroblasts, late fibroblasts show mitochondrial and proteostatic adaptations compatible with lower contractile output and sustained ECM servicing. Functionally, this profile supports long‐term scar homeostasis and a low‐inflammatory, low‐angiogenic niche (Table 1).

**TABLE 1:** Molecular and Functional Signatures of Fibroblast States During Cardiac Repair.

Aspect	Quiescent fibroblast	Myofibroblast	Matrifibrocyte
Identity/Lineage	Maintains lineage markers (*Tcf21*, *Pdgfra*, *Ddr2*)	Lineage identity downregulated (e.g., *Tcf21*↓)	Partially retained lineage identity (*Tcf21* low but detectable)
Contractile Machinery	Lacks *α*‐SMA; non‐contractile	Strongly expresses *α*‐SMA, *Tagln*, *Myh11* (stress fibres)	*α*‐SMA silenced; reduced stress fibres; transition to non‐contractile phenotype
ECM Synthesis/Remodelling	Basal *Col1a1*, *Col3a1*, *Mmp2* for maintenance	High *Acta2*, *Postn*, *Col1a1, Lox* for deposition and cross‐linking	*Comp*, *Chad*, *Thbs4* for matrix stabilization and scar maintenance
Secretory/ER Adaptation	Low secretory activity; basal ER homeostasis	Induced *Thbs1*, *Atf3*, *Hspa5* (stress response)	*Thbs4* enhances ER homeostasis
Signalling Regulators	Basal paracrine tone; Meox1, Id3 maintain quiescence	TGF‐*β*/*Smad2/3*↑, *IL‐11*↑, YAP/TAZ↑ (mechanical activation)	TGF‐*β* activity↓, *Smad7*↑, *Sfrp2*↑, *Wisp2*↑, *Notch3*↑ (activation–resolution transition)
Metabolism/Stress Response	Oxidative phosphorylation	Glycolytic reprogramming, ROS stress	Mitochondrial adaptation, ER stabilization
Transcriptional Control	*Tcf21*, *Meox1*, *Id3* (suppress activation)	SRF, MRTF‐A, *Smad3*, YAP/TAZ (drive activation)	*Smad7*, *Atf6α*, *Notch3*, *Wisp2* (terminal stabilization)
Functional Output	ECM homeostasis, low tension	ECM synthesis, contraction, fibrosis	ECM stabilization, low tension and sustained scar homeostasis
Morphology/Tension	Spindle‐shaped, relaxed	Spread, stress fibre–rich, high tension	Rounded or stellate, non‐contractile, low mechanical tension

Collectively, these findings indicate that MFCs or matrifibrocyte‐like fibroblasts act not only as structural stabilizers but also as cellular regulators of scar homeostasis, orchestrating the balance between matrix integrity, inflammation resolution and angiogenic restraint in the post‐injury heart. Having defined the structural and regulatory functions of MFCs, the next step is to delineate the molecular mechanisms that drive their differentiation from activated MFs.

## 5 Molecular and Transcriptional Regulation of Matrifibrocyte Differentiation

MFC differentiation is driven by an integrated molecular–transcriptional network that converts injury resolution into a stable fibroblast fate. Rather than a passive waning of MF activity, it reflects active reprogramming shaped by cytokine signalling, mechanical cues and ECM feedback. Emerging studies outline how canonical profibrotic cascades and stress‐adaptive programs converge to establish the terminal MFC phenotype. However, direct tests of how classical fibrosis pathways govern MFC formation remain limited; current insights are largely inferential, drawn from transcriptional trends and parallels to fibroblast activation.

### 5.1 The TGF‐*β*/Smad Axis: *Smad7* as a Restrainer of Matrifibrocyte Accumulation

The transition into the MFC state is finely tuned by the TGF‐*β* signalling axis, with the inhibitory *Smad7* acting as a critical brake on its excessive accumulation. Evidence from myofibroblast‐specific *Smad7* knockout (MFS7KO) models provides a clear functional link: *Smad7* deficiency significantly increases the density of *Comp* + MFCs within the mature infarct scar [40]. This expansion is further corroborated by the upregulated expression of canonical MFC genes, including *Comp* and *Chad*, in fibroblast populations lacking *Smad7*. The mechanism by which *Smad7* constrains MFC accumulation involves a dual inhibitory capacity. Primarily, it attenuates the canonical TGF‐*β*/*Smad2/3* signalling that drives profibrotic activation. Furthermore, *Smad7* interacts with and suppresses the receptor tyrosine kinase ErbB2 in a TGF‐*β*‐independent manner, thereby closing a major alternative pathway that would otherwise promote MFC expansion [40]. Therefore, *Smad7* does not block the MFC differentiation program per se but acts as a crucial regulatory node to prevent its over‐amplification. By tempering both TGF‐*β*‐dependent and ‐independent (ErbB2) signalling inputs, *Smad7* ensures that the generation of MFCs is proportionate to repair needs, thereby preventing the disproportionate MFC accumulation that contributes to adverse stiffening and dysfunctional remodelling.

### 5.2 Predicted Pathways Regulating Matrifibrocyte Differentiation: Mechanotransduction, Wnt Attenuation, and Matrix‐Derived Signalling

Although direct evidence is limited, transcriptional enrichment, timing and mechanistic analogies implicate several fibrosis‐linked pathways in MFC differentiation: mechanotransduction via Hippo–YAP/TAZ, attenuation of canonical Wnt by *Sfrp2*, matrix‐driven *Comp–Notch3* feedback, and ER‐stress adaptation through the *Thbs4–Atf6α* axis. Together, these modules likely integrate mechanical, metabolic and extracellular cues to stabilise the MFC state and terminate fibroblast contraction.

#### 5.2.1 Mechanotransduction (Hippo–YAP/TAZ Signalling)

The mechanical properties of the ECM profoundly influence fibroblast differentiation. Functional evidence strongly implicates mechanotransduction pathways, particularly the Hippo‐YAP/TAZ signalling axis, in this process. A key finding demonstrates that high substrate stiffness is a potent driver of the MFC phenotype in vitro, as culturing CFs on soft hydrogels (4.5 kPa) suppresses the expression of bone/cartilage ECM genes, while stiff substrates promote their upregulation [41]. Given that YAP/TAZ are exquisitely sensitive to mechanical cues and are activated upon engagement with stiff matrices, these findings position them as the likely molecular transducers converting the mechanical cue of stiffness into the transcriptional program that defines MFCs [42]. Furthermore, the observed upregulation of cartilage‐ and bone‐associated genes in MFCs shares intriguing parallels with the known role of YAP/TAZ in chondrogenic and osteogenic differentiation, suggesting a conserved regulatory module [43, 44]. However, direct genetic evidence linking fibroblast‐specific YAP/TAZ activity to the in vivo formation or function of MFCs remains lacking and represents a critical focus for future work.

#### 5.2.2 Wnt Signalling Attenuation and Sfrp2‐Mediated Fibrosis Resolution

In the dynamic course of cardiac repair, Wnt signalling plays a biphasic role—promoting fibrosis in the early reparative phase and likely being suppressed as the scar matures. Canonical Wnt/*β*‐catenin signalling is strongly activated following myocardial injury, driving fibroblast proliferation and MFs differentiation through the induction of pro‐fibrotic genes such as *Postn* and *Col1a1*. Persistent Wnt activity, however, sustains ECM synthesis and propagates pathological remodelling [45]. In contrast, transcriptomic profiling of late‐stage fibroblasts reveals a striking upregulation of *Sfrp2*, a potent extracellular antagonist of Wnt signalling, within the matrifibrocyte‐like population [35]. *Sfrp2* binds Wnt ligands and Frizzled receptors, thereby preventing *β*‐catenin activation and attenuating canonical Wnt transcriptional output. This suggests that matrifibrocyte‐like cell differentiation coincides with down‐modulation of canonical Wnt signalling, representing a molecular switch that terminates the fibrogenic program and consolidates scar maturation. Functionally, such Wnt inhibition could help restrain further collagen synthesis, stabilize ECM architecture, and promote the transition to a non‐proliferative, post‐mitotic fibroblast state. Nonetheless, this remains a predictive hypothesis derived from transcriptomic trends rather than direct experimental evidence. Future fibroblast‐specific Wnt perturbation studies will be essential to determine whether *Sfrp2*‐mediated Wnt attenuation is causally required for MFC formation or merely reflects a downstream consequence of repair resolution.

#### 5.2.3 *Comp–Notch3* Signalling and Matrix‐Dependent Feedback

In addition to mechanical regulation, recent findings have revealed an additional layer of control linking ECM composition to fibroblast fate through the Notch signalling pathway [46]. Acting through direct cell–cell contact, Notch controls mesenchymal differentiation and ECM remodelling via ligand–receptor interactions (Jagged1/2, DLL1/4 with Notch1–4) and downstream activation of HES/HEY family transcription factors. Notably, *Comp*, one of the defining markers of MFCs, has been shown to directly interact with and activate *Notch3* signalling in stromal cells [47]. In a recent study of ovarian cancer stroma, *Comp* enhanced *Notch3*–Jagged1 interactions and stimulated downstream transcriptional programs that promoted matrix production and epithelial‐to‐mesenchymal transition [47]. Although these findings were derived from tumour stroma, they offer mechanistic insight relevant to cardiac scar biology, where a similar *Comp*–*Notch3* feedback loop may strengthen ECM gene expression and stabilize the post‐mitotic fibroblast phenotype. Collectively, these observations raise the possibility that Notch signalling, traditionally viewed as a developmental pathway, may act as a context‐dependent regulator of MFC differentiation and maintenance, integrating matrix‐derived and metabolic cues to consolidate the terminal fibroblast state.

#### 5.2.4 *Thbs4*–Atf6*α* Signalling and ER Stress Adaptation

MFC emergence during late repair coincides with sustained *Thbs4* upregulation [35, 37], peaking as scars mature after inflammatory/proliferative fibroblasts wane, consistent with a terminal fibroblast feature [48]. *Thbs4* is one of the most highly enriched matricellular genes within the MFC cluster identified by single‐cell analyses, paralleling its known role in secretory stress adaptation [27]. Mechanistically, Lynch et al. showed *Thbs4* activates a protective ER stress pathway by directly binding to *Atf6α*'s luminal domain, promoting *Atf6α* cleavage, nuclear translocation and induction of chaperone genes (BiP, Calreticulin, PDI). This *Thbs4–Atf6α* axis expands ER folding capacity and enhances protein secretion, protecting stressed cardiomyocytes from ER stress during injury. Since MFCs sustain high ECM production yet remain non‐apoptotic and quiescent, *Thbs4*‐mediated *Atf6α* activation may similarly operate in these cells to maintain ER homeostasis under chronic secretory load. This adaptive mechanism could enable long‐term survival of matrix‐secreting fibroblasts within the mature scar while preventing ER stress–induced apoptosis. However, this remains a hypothesis based on *Thbs4* transcriptional enrichment rather than direct functional evidence. Future studies examining fibroblast‐specific *Thbs4* gain‐ or loss‐of‐function and assessing *Atf6α* signalling in post‐infarction scars are essential to determine if this adaptive ER stress pathway contributes to MFC differentiation or maintenance.

**FIGURE 2:**
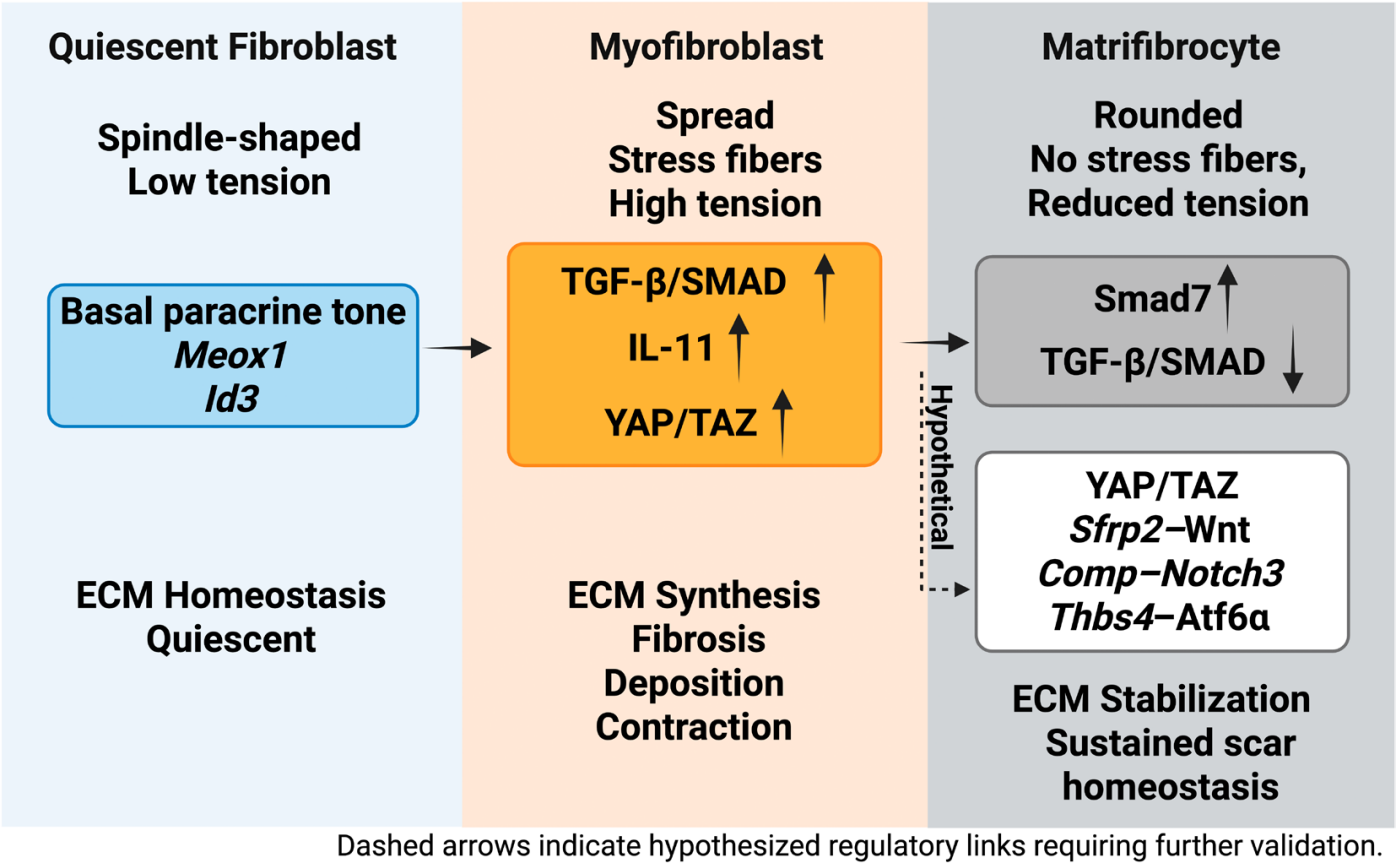
Molecular and transcriptional regulation of Matrifibrocyte differentiation. CFs transition from a quiescent, low‐tension state to activated MFs driven by TGF‐*β*/SMAD, IL‐11 and YAP/TAZ signalling. Terminal differentiation into MFCs is associated with *Smad7* upregulation and TGF‐*β* suppression, along with proposed regulatory modules, including YAP/TAZ reprogramming, *Sfrp2*–Wnt modulation, *Comp*–*Notch3* signalling and *Thbs4–Atf6α*–mediated ER stress adaptation, which collectively stabilize the ECM and maintain scar homeostasis.

Together, emerging evidence points to a convergent control system in which mechanotransduction (Hippo–YAP/TAZ), *Sfrp2*‐mediated Wnt restraint, matrix‐driven Notch signalling (*Comp–Notch3*), and adaptive ER‐stress responses (*Thbs4–Atf6α*) coordinate the shift from activated MFs to terminal MFCs. Acting in parallel and sequentially, these pathways relay mechanical, paracrine and intracellular cues to halt fibroblast activation, stabilise ECM architecture and maintain scar homeostasis. Defining their crosstalk and context‐dependent tuning across injury, aging and disease is a key priority that may enable therapeutic modulation of the MFC program to resolve fibrosis without compromising scar stability (Figure 2).

## 6 Disease Universality and Heterogeneity

The biological significance of MFCs extends beyond the context of myocardial infarction, where they were first defined. Emerging evidence from diverse pathological models indicates that the MFC state represents a common, yet potentially heterogeneous, endpoint in cardiac fibrotic remodelling.

### 6.1 Prototypical Disease: Myocardial Infarction

Myocardial infarction is the canonical setting in which MFCs were first defined as a persistent, lineage‐traced fibroblast population essential for long‐term scar integrity [14]. Their appearance after inflammatory and proliferative phases marks scar maturation and resolution in ischemic injury. Transcriptomic and functional data indicate that MFCs consolidate extracellular matrix organization, maintain tensile strength, and preserve ventricular geometry once myofibroblast contraction subsides. Meta single‐cell analyses further refine this view. Ke et al. reconstructed fibroblast trajectories across 12 post‐infarction datasets and identified reparative CFs dominating early healing and MFCs emerging during subacute to chronic stages, approximately days 14 to 28, enriched in the infarct zone and expressing *Comp*, *Thbs4*, *Chad* and *Sfrp2*, consistent with Fu et al. [14, 49]. Collectively, findings from both lineage‐tracing and high‐resolution single‐cell studies firmly establish myocardial infarction as the canonical setting in which the MFC state emerges, linking its differentiation to the transition from active repair to long‐term scar maintenance. In infarction, late fibroblasts also show anti‐inflammatory bias linked to *Sfrp2* [38], *Wisp2* [50] and *Smad7* [51], together with matrix‐coupled paracrine or intercellular feedback that reinforces scar maintenance.

### 6.2 Non‐Ischemic Remodelling and Heart Failure

The MFC phenotype also arises during chronic non‐ischemic remodelling driven by pressure overload and neurohormonal activation. In angiotensin II infusion and transverse aortic constriction (TAC), fibroblasts upregulate matrifibrocyte‐associated markers *Comp*, *Chad*, *Thbs4*, *Sfrp2* and downregulate *Acta2* [35, 36]. *Thbs4* is strongly induced by pressure overload and linked to ER‐stress adaptation during TAC‐induced hypertrophy, suggesting shared stress‐response mechanisms in non‐ischemic MFC differentiation [48, 52]. Neurohormonal stimulation with chronic angiotensin II similarly provokes interstitial fibrosis with *Cilp*, *Thbs4*, *Sfrp2*, *Comp* induction and loss *of Acta2*, and pseudotime indicates a *Cilp* ^+^ to *Thbs4* ^+^ shift consistent with emergence of an ECM‐rich, non‐contractile population [36]. In the pressure‐loaded right ventricle after pulmonary artery banding, early activation is followed by a late matrifibrocyte‐like transition [53]. At 1 week *Postn*, *Thbs4*, *Ltbp2*, *Meox1* increase, whereas by 6 weeks *Cthrc1* declines and *Comp* rises, paralleling the reparative‐to‐MFC switch. Together, these findings indicate that chronic hemodynamic or neurohormonal stress elicits a conserved fibroblast trajectory with early activation followed by stabilization into a *Thbs4* ^+^ */Sfrp2* ^+^ */Comp* ^+^ matrifibrocyte‐like state. In loaded territories, enrichment of these late states is accompanied by ER proteostatic adaptation consistent with the *Thbs4‐ATF6α* axis, attenuation of Wnt signalling via *Sfrp2*, and matrix‐coupled paracrine or intercellular feedback that reinforces stabilization. This terminal program helps maintain matrix integrity under sustained load, but its persistence may also promote pathological stiffening in non‐ischemic heart disease.

### 6.3 Valvular Heart Disease

**FIGURE 3:**
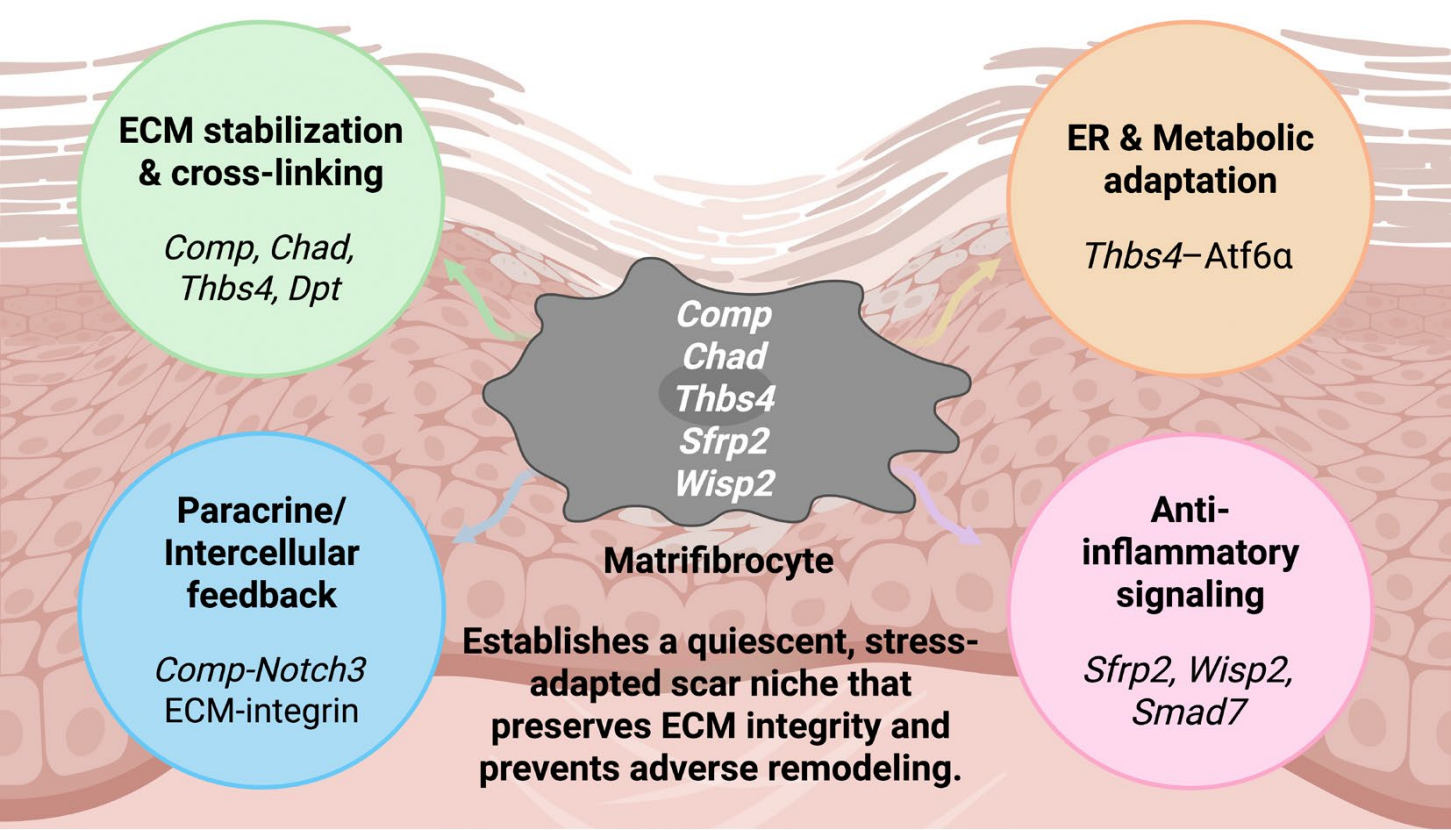
Functional roles of MFCs in the mature scar microenvironment. Matrifibrocytes deploy coordinated programs for extracellular‐matrix stabilization and cross‐linking (*Comp*, *Chad*, *Thbs4*, *Dpt*), ER and metabolic adaptation via *Thbs4–Atf6α*, anti‐inflammatory signalling (*Sfrp2*, *Wisp2*, *Smad7*), and paracrine or intercellular feedback including *Comp–Notch3* and ECM–integrin coupling. Together these modules establish a quiescent, stress‐adapted scar niche that preserves ECM integrity and limits adverse remodelling.

The concept of the matrifibrocyte extends to valvular pathology. Single‐cell and spatial analyses show that valve interstitial cells (VICs) undergo disease‐specific differentiation programs paralleling cardiac fibroblasts during scar maturation. In calcific aortic valve disease (CAVD), single‐cell profiling of bicuspid valves identified multiple VIC subpopulations with distinct trajectories [54]. *Comp* ^+^ and *Postn* ^+^ VICs occupied terminal branches enriched for extracellular‐matrix synthesis and ossification pathways, exhibited a non‐contractile, matrix‐stabilizing phenotype reminiscent of MFCs, and localized to fibrotic and calcified regions. Although not labelled ‘matrifibrocytes’, these *Comp* ^+^ */Postn* ^+^ VICs likely represent a valvular counterpart contributing to ECM cross‐linking and calcific stiffening. A recent single‐cell atlas of the human tricuspid valve provided direct evidence for matrifibrocyte‐driven valvular fibrosis [55]. In functional tricuspid regurgitation, VICs transitioned toward myofibroblast‐like and matrifibrocyte‐like states; the latter expressed high Comp and localized to dense interstitial fibrosis. Mechanistically, *Comp* engaged *CD47* to enhance fibroblast–matrix adhesion and sustain profibrotic signalling, forming a self‐reinforcing fibrotic niche. These studies indicate that matrifibrocyte‐like VICs represent a conserved terminal fibroblast fate across valves and myocardium: by maintaining ECM integrity and tensile strength they stabilize chronically stressed valves, yet persistence and enhanced cross‐linking may promote pathological stiffening and progressive dysfunction. Overall, MFC differentiation appears to be a conserved endpoint of fibroblast activation across myocardial infarction, pressure overload, and valvular disease; defining the governing pathways may enable therapies that modulate fibrosis while preserving scar stability (Figure 3).

## 7 Translational Implications and Future Directions

Recognition of the MFC reframes fibrosis as a state trajectory that culminates in a differentiated endpoint preserving scar integrity. Because this state appears tunable, therapeutic strategies can steer fibroblast fate to balance early mechanical stabilization with later functional recovery.

### 7.1 The Duality of Therapeutic Strategy: A Challenge of Precision Control

A core therapeutic dilemma is the opposing needs at different stages of heart disease. After acute myocardial infarction, preventing lethal complications (e.g., ventricular rupture) is paramount; in this context, promoting the timely transition of MFs to MFCs may accelerate scar maturation and mechanical stabilization. Supporting this, Mullany et al. showed that the small‐molecule MCB‐613, a potent stimulator of SRC‐1/2/3, markedly improved post‐MI function when administered within hours of ischemia. Single‐cell RNA‐seq indicated reduced differentiation of *Acta2* ^+^ MFs and dampened IL‐1 signalling during the acute phase, while the chronic phase showed enrichment of Comp^+^ fibroblasts and increased *Wisp2* and *Sfrp2*—hallmarks of a matrifibrocyte‐like state [56]. Concomitantly, pro‐inflammatory macrophages were suppressed and Tsc22d3^+^ reparative macrophages expanded, creating a pro‐healing, anti‐inflammatory milieu that stabilized scar architecture. These findings suggest that pharmacologic SRC stimulation with MCB‐613 can hasten the shift from MF activation to MFC stabilization, preventing adverse remodelling while preserving infarct integrity [56].

Beyond SRC modulation, Burke et al. demonstrated that the ionophore salinomycin prevents pathological fibrosis and maladaptive remodelling in angiotensin II–induced hypertension and post‐infarction models [57]. Mechanistically, salinomycin suppresses p38/MAPK and Rho/ROCK pathways, limiting MF contractility and extracellular‐matrix deposition. Transcriptomic profiling further showed normalization of senescence‐associated and matrifibrocyte‐related markers, restoration of a more quiescent fibroblast phenotype, and reductions in collagen accumulation and ventricular stiffening. These data position salinomycin as a pharmacologic prototype that resets fibroblast activation dynamics and promotes functional scar homeostasis [57].

In contrast to pharmacologic stabilization, regenerative reprogramming seeks to reverse established fibrosis by converting fibroblasts into cardiomyocytes. Tani et al. reported that inducible expression of *Mef2c/Gata4/Tbx5/Hand2* (MGTH) in *Tcf21* ^+^ CFs improved ventricular function and reduced scar burden in chronic MI [58]. Single‐cell RNA‐seq revealed regression of Comp^+^/Thbs4^+^ matrifibrocyte‐like clusters (CF5/CF6; enriched for cartilage/ossification genes such as *Comp*, *Ecrg4*, *Cytl1*, *Thbs4*, *Chodl*, *Tnmd*) alongside re‐emergence of quiescent *Dpt* ^+^ */Lpl* ^+^ fibroblasts [58]. These findings indicate that MFC differentiation is not strictly irreversible and that fibroblast terminal states are actionable. Small molecules (e.g., MCB‐613, salinomycin) can accelerate MFC maturation or restrain maladaptive activation to stabilize scars, whereas MGTH‐based reprogramming can suppress or reverse the MFC program, together positioning MFCs as a tractable target for precision control of chronic fibrotic remodelling.

### 7.2 Outstanding Questions and Future Directions

**FIGURE 4:**
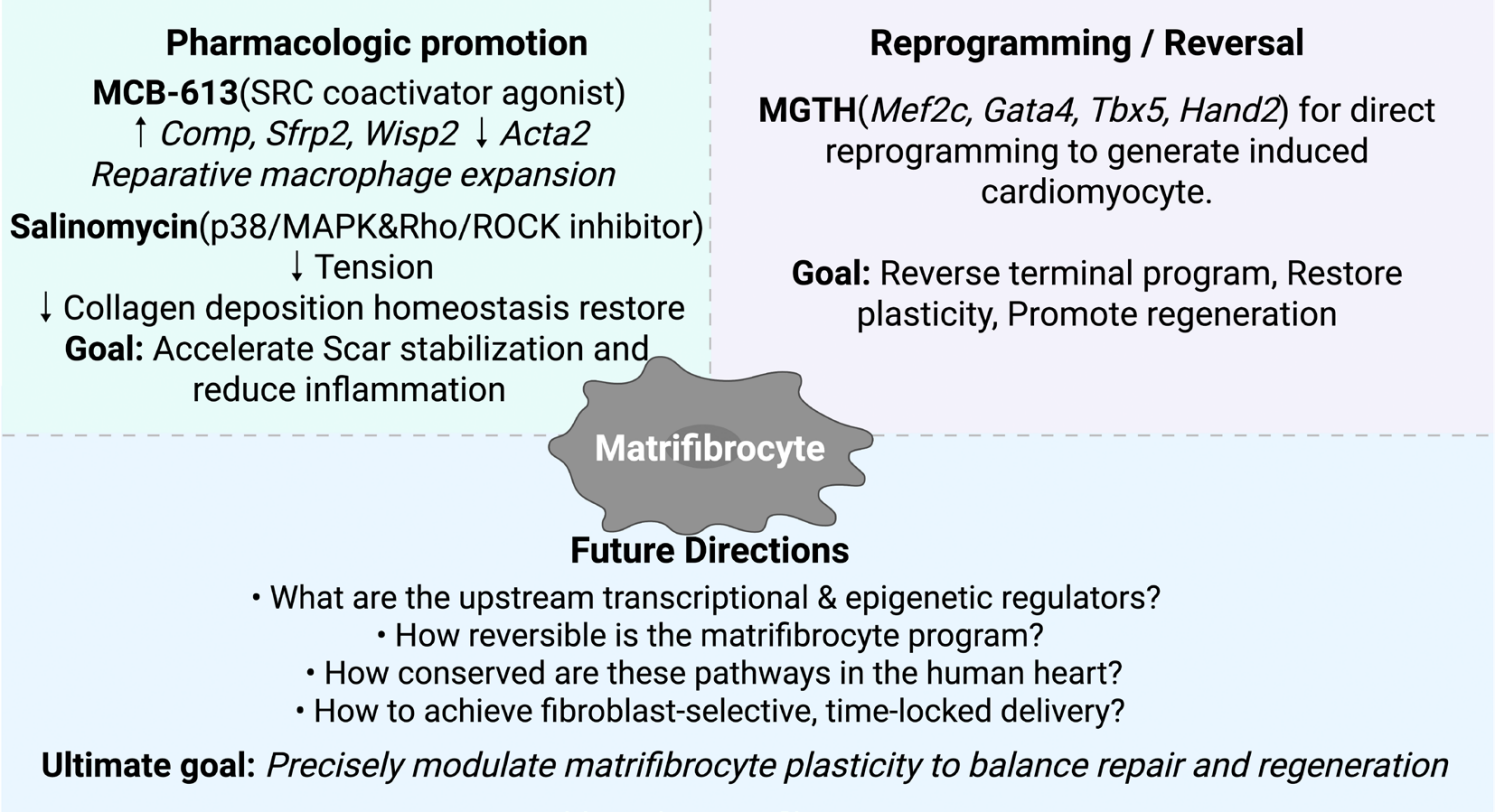
Therapeutic and research framework centered on the matrifibrocyte state. The MFC is a pharmacologically and transcriptionally modifiable fibroblast state linking scar stabilization and regeneration. *Left*: Pharmacologic activation (e.g., MCB‐613, salinomycin) enhances MFC maturation, stabilizes ECM, and reduces inflammation. *Right*: Reprogramming strategies (e.g., MGTH factors) reverse terminal differentiation and promote cardiomyocyte regeneration. *Bottom*: Outstanding questions concern upstream regulation, reversibility, and precise targeting of MFCs to balance repair and recovery.

Although remarkable progress has been made in defining the origin, phenotype, and functional role of MFCs, several critical questions remain unanswered. At the molecular level, the upstream transcriptional and epigenetic regulators that initiate and stabilize the MFC program are still poorly understood. Elucidating these master switches will be essential for developing strategies that can selectively steer fibroblast fate toward repair or reversal. Another challenge lies in bridging insights across species and disease contexts. Comparative analyses of murine and human datasets have revealed conserved MFC signatures, yet the timing, abundance, and signalling dependencies of this cell state may vary depending on the type and chronicity of injury. Integrating cross‐species single‐cell and spatial transcriptomic data, alongside experimental validation in human‐derived cardiac organoids or ex vivo tissue systems, will be key to establishing mechanistic continuity from model organisms to clinical disease. The plasticity of the MFC state also remains incompletely defined. While traditionally viewed as terminally differentiated, emerging evidence from reprogramming studies suggests that matrifibrocyte‐like fibroblasts may retain latent potential for regression or lineage conversion under specific transcriptional or mechanical cues. Defining the molecular checkpoints that permit or restrict such reversibility will have profound implications for antifibrotic therapy. Finally, the translation of these insights into therapy will depend on the development of precision targeting strategies capable of manipulating MFCs within diseased myocardium without disrupting their homeostatic functions in other connective tissues such as valves and tendons. Achieving this will require fibroblast‐specific delivery platforms, temporally controlled intervention windows, and integrative modelling of cardiac remodelling. Collectively, addressing these challenges will move the field from descriptive characterization to functional control. A deeper understanding of the determinants governing MFC formation, maintenance, and reversibility may ultimately enable precise modulation of cardiac fibrosis—preserving scar integrity while restoring myocardial compliance and performance (Figure 4).

## 8 Concluding Remarks

In summary, the recognition of MFCs as a distinct and regulatable fibroblast state has reframed our understanding of cardiac fibrosis, from an inevitable endpoint of injury to a dynamic and adaptable process. Integrating insights from lineage tracing, single‐cell profiling, and translational modelling now allows a unified view of how fibroblast activation, stabilization, and potential regression are orchestrated across disease contexts. As mechanistic and technological advances continue to converge, strategies that precisely modulate MFC formation and persistence may ultimately enable the heart to repair without scarring and to remodel without failing.

## Author Contributions

**Zhentao Zhang:** writing – original draft, visualization, writing – review and editing. **Hua Zhu:** writing – review and editing.

## Funding

This work was supported by the National Institutes of Health (R01AG071676, R01AR067766, R01HL153876), American Heart Association (23CDA1045959, 23TPA1142638).

## Conflicts of Interest

The authors declare no conflicts of interest.

## Data Availability

Data sharing not applicable to this article as no datasets were generated or analysed during the current study.
